# Na^^+^^/K^^+^^-ATPase regulates the K^^+^^/Na^^+^^ homeostasis in the intertidal macroalgae, *Neoporphyra haitanensis*, in response to salt stress

**DOI:** 10.3389/fpls.2022.1040142

**Published:** 2023-01-04

**Authors:** Qi Chen, Kai Xu, Yan Xu, Dehua Ji, Changsheng Chen, Chaotian Xie, Wenlei Wang

**Affiliations:** ^1^ Fisheries College, Jimei University, Xiamen, China; ^2^ Fujian Engineering Research Center of Aquatic Breeding and Healthy Aquaculture, Fujian Development and Reform Commission, Xiamen, China; ^3^ Key Laboratory of Healthy Mariculture for the East China Sea, Ministry of Agriculture, Xiamen, China

**Keywords:** K^+^/Na^+^ homeostasis, Na^+^/K^+^-ATPase, Na^+^/H^+^ antiporter, intertidal macroalgae, salt stress

## Abstract

In plants under hypersaline stress, the main transporter that extrudes sodium ions (Na^^+^^) is the Na^^+^^/H^^+^^ antiporter SOS1. Different from land plants, the intertidal macroalgae, *Neopyropia/Neoporphyra* contains an animal-type Na^^+^^/K^^+^^-ATPase as well as the SOS1 system. However, the contribution of Na^^+^^/K^^+^^-ATPase to the K^^+^^/Na^^+^^ homeostasis of intertidal macroalgae remains unclear. In this study, we analyzed the function of Na^^+^^/K^^+^^-ATPase in the response of *Neoporphyra haitanensis* to salt stress from the perspective of ion transport dynamics. Both the transcript level of *NhNKA2* and enzyme activity of Na^^+^^/K^^+^^-ATPase increased in the early response of *N. haitanensis* thalli to hypersaline stress. Addition of ouabain, an inhibitor of Na^^+^^/K^^+^^-ATPase, resulted in Na^^+^^ accumulation in the cells, severe K^^+^^ leakage from the thalli, and then remarkably disturbed the K^^+^^/Na^^+^^ homeostasis in *N. haitanensis* thalli. This disruption might induce a significant decrease in photosynthesis and a severe oxidative damage in thalli. Accordingly, these results suggested that the important role of Na^^+^^/K^^+^^-ATPase in the resistance of intertidal macroalgae to hypersaline stress, and shed light on the diversity of K^^+^^/Na^^+^^ homeostasis maintenance mechanisms in plants.

## 1 Introduction

Crop losses due to soil salinity are a growing threat to global agriculture, with increased salinity causing 1.5 million ha of farmland to become unsuitable for agriculture, and 31 billion USD in economic losses annually (Zhu, 2016; Isayenkov and Maathuis, 2019). Studies on the mechanisms by which plants adapt to and tolerate salinity are useful for breeding new varieties with improved salt tolerance. Macroalgae growing in the intertidal zone are subject to severe salinity fluctuations owing to periodic dehydration during low tides and rehydration during high tides (Blouin et al., 2011; Wang et al., 2019). To cope with the challenges of this harsh environment, intertidal macroalgae have evolved stronger tolerance to salinity stress than have terrestrial plants and aquatic algae. Therefore, intertidal macroalgae are ideal biological models to explore the salinity stress resistance mechanism of algae and higher plants.

Excessive Na^^+^^ accumulation in plant cells and tissues has toxic effects. High levels of salt stress can inhibit growth and energy metabolism, and cause premature aging and even death (Zhang et al., 2020). Under salt stress, Na^^+^^ partially replaces K^^+^^, which is crucial for maintaining metabolism and normal growth. This increased Na^^+^^ level limits the influx of K^^+^^ and results in an imbalance in the intracellular K^^+^^/Na^^+^^ ratio. To maintain K^^+^^/Na^^+^^ homeostasis in the cytoplasm under salt stress, plants limit the accumulation of Na^^+^^ and the loss of K^^+^^ (Hussain et al., 2021). Eukaryotes have two major Na^^+^^ efflux systems: the salt overly sensitive (SOS) system, which is a universally conserved salt tolerance pathway in plants; and the Na^^+^^/K^^+^^-ATPase system in bacteria, fungi, moss, and mammals (Nakayama et al., 2010; Jacobs et al., 2011; Dabravolski and Isayenkov, 2021). The function of SOS1 relies on the plasma membrane (PM)-localized H^^+^^-ATPase that hydrolyzes adenosine triphosphate (ATP) to generate energy and pumps H^^+^^ out of the cytoplasm to generate an electrochemical gradient (Qiu et al., 2002; Haruta and Sussman, 2012; Yang et al., 2019). Our previous studies demonstrated that high salinity activates SOS1 in the marine macroalga *Neoporphyra haitanensis*, resulting in the expulsion of excess Na^^+^^ to maintain low intracellular Na^^+^^ levels. This process also prevents Na^^+^^-induced plasma membrane depolarization and avoids K^^+^^ leakage from outward rectifying K^^+^^ channels (KORCs), thereby maintaining a high K^^+^^/Na^^+^^ ratio (Chen et al., 2019; Wang et al., 2020). Interestingly, the thalli of marine red alga *Neoporphyra*/*Neopyropia* (previously named *Porphyra*/*Pyropia*) genus also harbor Na^^+^^/K^^+^^-ATPases that are very similar to animal Na^^+^^/K^^+^^-ATPases (Barrero-Gil et al., 2005; Kishimoto et al., 2013; Kumari and Rathore, 2020). The Na^^+^^/K^^+^^-ATPase is an energy-transporting ion pump located on the cytoplasmic membrane. For each ATP molecule consumed, three Na^^+^^ are pumped out and two K^^+^^ are pumped in against the electrochemical gradient (Gadsby et al., 2012). Therefore, compared with SOS1, Na^^+^^/K^^+^^-ATPase can more directly and efficiently regulate K^^+^^/Na^^+^^ homeostasis under salt stress.

In an evolutionary context, Na^^+^^/K^^+^^-ATPases can be divided into animal and fungal types, flowering plants are generally considered to lack Na^^+^^/K^^+^^-ATPases (Pedersen et al., 2012; Dabravolski and Isayenkov, 2021). Genomic analyses revealed that genes encoding Na^^+^^/K^^+^^-ATPases were lost during evolution and land settlement, suggesting that Na^^+^^/K^^+^^-ATPases might exist in eukaryotes living in high-salinity marine environments (Garciadeblas et al., 2001; Pedersen et al., 2012). Such as, the intertidal alga *Neopyropia yezoensis* has two Na^^+^^/K^^+^^-ATPase genes, *PyKPA1* and *PyKPA2*. *PyKPA1* preferentially expressed in sporophytes, whereas *PyKPA2* specifically expressed in gametophytes (Uji et al., 2012; Kishimoto et al., 2013). We previously also screened two genes encoding Na^^+^^/K^^+^^-ATPase (*NhNKA1* and *NhNKA2*) in *N. haitanensis* genome (unpublished). Additionally, Kishimoto et al. (2013) found that *PyKPA1* could increase salt tolerance of rice via comparison of biomass level between control and *PyKPA1*-overexpressing rice seedlings. Nevertheless, the specific physiological functions of Na^^+^^/K^^+^^-ATPases in maintaining K^^+^^/Na^^+^^ homeostasis in intertidal macroalgae under salt stress are still unknown.

Non-invasive micro-test technology (NMT) can be used to detect the dynamic flow of ions/molecules in real time, and can provide accurate information about the regulation of ion balance in plant tissues and cells (Han et al., 2022). The NMT measurement method is simple, convenient, and fast. In recent years, NMT has become an important method to study ion transport across membranes, and studies using this technique have improved our understanding of the mechanism of ionic homeostasis in plants exposed to hypersaline stress (Wu et al., 2019). It has also been used to investigate the role of SOS1 in the maintenance of K^^+^^/Na^^+^^ dynamic homeostasis in *N. haitanensis* (Chen et al., 2019; Wang et al., 2020). In this study, the net fluxes of Na^^+^^ and K^^+^^ were determined by NMT in the presence of an Na^^+^^/K^^+^^-ATPase inhibitor, ouabain, and under salt stress. The functions of Na^^+^^/K^^+^^-ATPase and SOS1 in the response to salt stress were compared from the perspective of ion transport dynamics. The results of this study provide new insights into how intertidal macroalgae maintain K^^+^^/Na^^+^^ homeostasis in response to salt stress.

## 2 Materials and methods

### 2.1 Culture and stress treatment of *N. haitanensis*

The *N. haitanensis* strain Z-61 was screened and purified by the Laboratory of Germplasm Improvements and Applications of Pyropia at Jimei University, Fujian Province, China. The thalli were cultured in tanks with Provasoli’s enrichment solution (PES) at 21°C ± 1°C under cool-white fluorescent illumination (50–60 μmol photons m^−2^ s^−1^) with a 12:12 h light:dark (L:D) photoperiod. The PES medium was refreshed every 2 days. Previous studies have determined that *N. haitanensis* thalli can adapt to 100‰ saline stress, and that 110‰ is a sub-lethal concentration, as thalli can recover from this treatment if they are transferred to normal seawater (Chen et al., 2019). Therefore, with normal seawater as the control (CK, 30‰), thalli (15 ± 1 cm) were randomly selected and cultured in aerated flasks under hypersaline stress (100‰ and 110‰) for 5 min, 10 min, 15 min, 30 min, 1 h, 2 h, 4 h, and R240 (recovery for 240 min in normal seawater following the salt stress treatment). In subsequent experiments, three biological replicates were established for each group.

### 2.2 Purification of total RNA and synthesis of cDNA

Total RNA was extracted from *N. haitanensis* thalli using an E.Z.N.A Plant RNA Extraction Kit (Omega Bio-tek, Norcross, GA, USA). The quality of total RNA was checked by 1% gel electrophoresis. The OD_260_ nm and OD_280_ nm values were measured using an ultraviolet spectrophotometer, and the RNA concentration was calculated to judge its purity. The RNA was reverse-transcribed to synthesize cDNA with a Prime Script RT reagent kit (TaKaRa, Dalian, China), and diluted 10-fold for subsequent qRT-PCR analyses.

### 2.3 Quantitative real-time PCR analysis

*NhUBC* encoding ubiquitin conjugating enzyme (Li et al., 2014) was used as the reference gene to normalize the transcript levels of *NhNKA1*, *NhNKA2*, and *NhSOS1*, encoding the *N. haitanensis* Na^^+^^/K^^+^^-ATPase and Na^^+^^/H^^+^^ antiporter, respectively, under different treatments. The primers used to amplify three genes were as follows: *NhNKA1* (forward: CGTCTGCCCAGCCGAACTTT; reverse: GGTCTGCGACACGCCATTGAT), *NhNKA2* (forward: TCCTCTGCTTTATCGGCTT; reverse: AACTTTTCCATCGTCTTCTCG), and *NhSOS1* (forward: CACGACTTTCTCGTCGCACA; reverse: GGGAGAAGATTCCGTTGGCG). The qRT-PCR program was as follows: 95 °C for 30 s, 40 cycles of 95 °C for 5 s, and 54.3 °C for 30 s.

### 2.4 Determination of Na^^+^^/K^^+^^-ATPase activity

The treated thalli were collected and weighed, the impurities on the surface of the samples with cold Phosphate Buffered Saline buffer and homogenized in ice bath according to the proportion of 1mL extraction buffer added to 0.1 g tissue. Finally, absorbance is measured at 660 nm by colorimetric method (Sun et al., 2021). Na^^+^^/K^^+^^-ATPase catalyzes the hydrolysis of adenosine triphosphate (ATP) to generate adenosine diphosphate (ADP) and inorganic phosphate (Pi), and the Pi content can reflect enzyme activity. One unit of enzyme activity (U) was defined as the amount of Na^^+^^/K^^+^^-ATPase producing 1 μmol of Pi by the hydrolysis of ATP per gram tissue per hour.

### 2.5 Chlorophyll fluorescence

The optimal quantum yield (F_v_/F_m_) reflects the maximum quantum yield of photosystem II (PSII). To determine the effects of salt stress and various inhibitors on the thalli of *N. haitanensis*, the chlorophyll fluorescence of PSII was measured with a portable pulse amplitude modulation fluorometer (Diving-PAM, Walz, Effeltrich, Germany). Intrinsic fluorescence (F_0_) was measured for thalli after 10 minutes of dark adaptation. A saturated flash (5000 μmol photons m^−2^s^−1^, 800 ms) was then applied to the thalli to obtain maximum fluorescence (F_m_). The variable fluorescence (F_v_) was recorded as the difference between F_m_ and F_0_, and then F_v_/F_m_ was calculated (Schreiber, 2004).

### 2.6 Pharmacological experiments

We used ouabain as a Na^^+^^/K^^+^^-ATPase inhibitor. The final concentration of ouabain was 100 nM. The concentration of amiloride (a SOS1 inhibitor) was 100 μM as described in our previous study (Chen et al., 2019).

### 2.7 Ion flux measurements

According to the F_v_/F_m_ results, the salt stress treatment time was set to 15 min and salinity was set to 100‰ for subsequent analyses of Na^^+^^ and K^^+^^ fluxes. The net fluxes of Na^^+^^ and K^^+^^ were measured using an NMT system (Younger USA LLC, Amherst, MA, USA). Before measuring ion fluxes, the microelectrodes were calibrated with standard solutions and the thalli were immersed in the measurement solution for 5 min. The standard and measurement solutions were based on those described by (Chen et al., 2019). The standard solutions were as follows: 500/50 mM NaCl, 2.0 mM, NaHCO_3_, 10/1 mM KCl, 0.1 mM Na_2_SO_4_ and 0.1 mM CaCl_2_, 0.3 mM MES, pH 7.5/8.5. The measurement solutions were as follows: 360 mM NaCl, 2.0 mM NaHCO_3_, 8.0 mM KCl, 0.1 mM Na_2_SO_4_ and 0.1 mM CaCl_2_, 0.3 mM MES, pH 8.1.

### 2.8 Measurement of Na^^+^^ and K^^+^^ contents

To avoid the influence of external Na^^+^^ and K^^+^^, the thalli were quickly rinsed with sorbitol isotonic solution before extracting Na^^+^^ and K^^+^^. Each thallus sample (0.01–0.02 g dry weight) was pulverized, nitrified with 5 mL of HNO_3_ at 50°C for 2 h, and then nitrified overnight in a fume hood. The Na^^+^^ and K^^+^^ contents were determined using an induced coupled plasma-optical emission spectrometer (ICP-OES, Prodigy 7, Leeman, Hudson, NH, USA) as described by (Chen et al., 2019). The Na^^+^^ and K^^+^^ concentrations were determined from standard calibration curves.

### 2.9 Determination of reactive oxygen species and malondialdehyde contents

The contents of reaction oxygen species (ROS, specifically hydrogen peroxide (H_2_O_2_) and superoxide ( ${\text{O}}_{2}^{-}$ )) and malondialdehyde (MDA) in *N. haitanensis* were determined at different times under hypersaline stress and inhibitor treatments. The specific determination methods were as described elsewhere (Zhang et al., 2011).

### 2.10 Statistical analyses

Data were analyzed by one-way analysis of variance (ANOVA), with a significance level of 0.05. All reported *P-*values are based on one-way ANOVA followed by least significant difference (LSD) tests unless otherwise stated.

## 3 Results

### 3.1 Changes in *NhNKA* transcript levels and NhNKA enzyme activity under hypersaline stress

**Figure 1:**
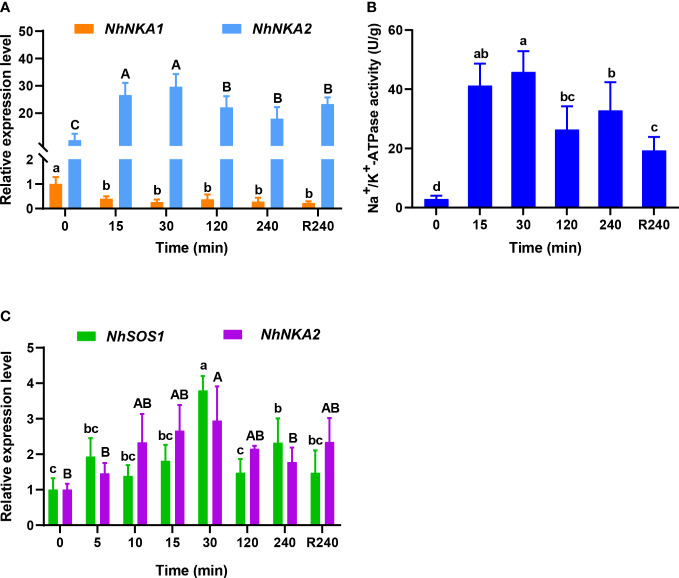
Transcript levels of Na^+^/K^+^-ATPase (NKA), Na^+^/H^+^ antiporter (SOS1) and enzyme activity of NhNKA in *N. haitanensis* at different times under hypersaline stress. **(A)** Transcript levels of *NhNKA1* and *NhNKA2* under 100‰ hypersaline stress. **(B)** Enzyme activity of NhNKA under 100‰ hypersaline stress. **(C)** Transcript levels of *NhSOS1* and *NhNKA2* in the very early phase of 100‰ hypersaline stress. R240: 240 min recovery from salt stress in normal seawater (30‰). Values shown are mean ± standard error of the mean (*n* = 3). Different letters indicate significant differences (*P <* 0.05).

Under 100‰ hypersaline stress, the transcript level of *NhNKA1* did not increase in gametophytes thalli (**Figure 1A**), but the transcript level of *NhNKA2* significantly increased at the initial stage of stress (15 min), to about 2.67 times that in the CK, respectively (**Figure 1A**). After recovery in normal seawater for 240 min, the transcript level of *NhNKA2* had decreased slightly but was still significantly higher than that at 0 h, and was about 3.24 times that in CK (**Figure 1A**). The enzyme activity of NhNKA also increased significantly after 100‰ hypersaline treatment (**Figure 1B**). It peaked at 30 min of hypersaline treatment, at 15.71 times that in the CK (**Figure 1B**). After recovery in normal seawater for 240 min, NhNKA activity had decreased slightly but was still significantly higher than that at 0 h, and was 6.63 times that in the CK (**Figure 1B**). In addition, the transcript levels of both *NhNKA2* and *NhSOS1* were up-regulated in the very early phase (5 min) of hypersaline stress (**Figure 1C**).

### 3.2 Effects of ouabain on photosynthesis in *N. haitanensis*

**Figure 2:**
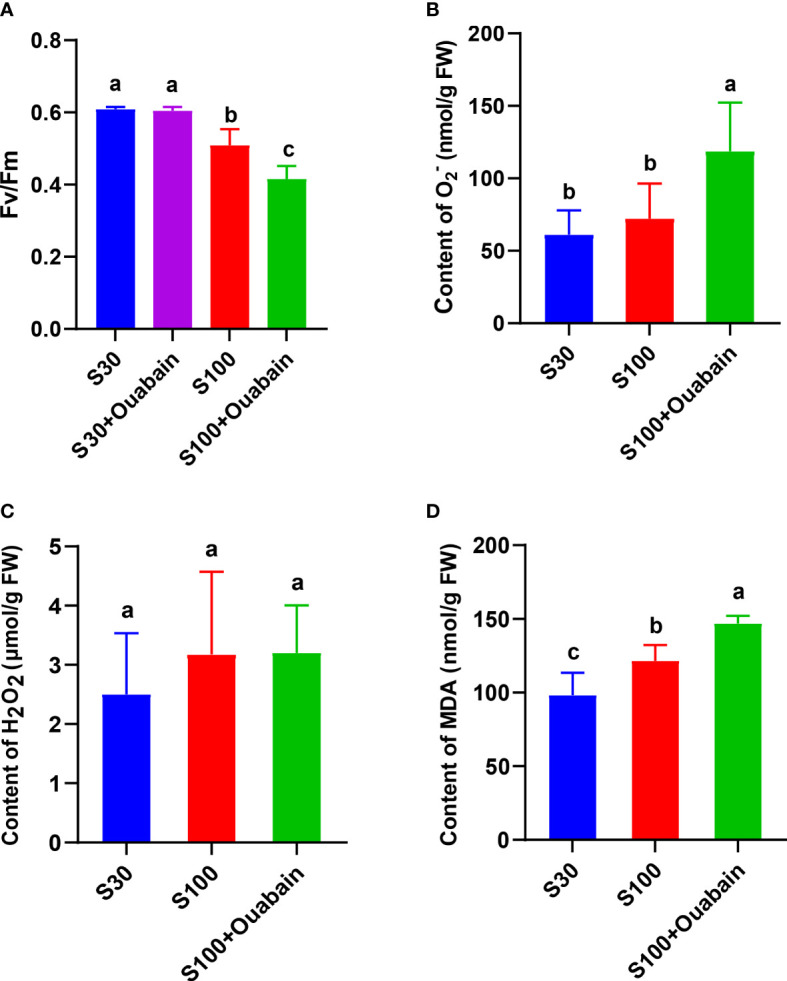
Effects of hypersaline stress (100‰, 15 min) and ouabain on physiological indexes of *N. haitanensis.* **(A)** Variations in photosynthetic parameters and optimum quantum yield (F_v_/F_m_) of *N. haitanensis* under hypersaline stress and in the presence of ouabain. O_2_− content **(B)**, H_2_O_2_ content **(C)** and MDA content **(D)** of *N. haitanensis* under hypersaline stress and in the presence of ouabain. FW: fresh weight of thalli. S30: control salinity (30‰). S100: hypersaline stress (100‰). Values shown are the mean of three thalli. Bars represent the standard error of the mean (*n* = 3). Different letters indicate significant differences among treatments (*P<* 0.05).

In normal seawater, the F_v_/F_m_ of *N. haitanensis* thalli was not significantly different between a control group (no inhibitor) and a group treated with 100 nM ouabain (Na^^+^^/K^^+^^-ATPase inhibitor) for 15 min (**Figure 2A**). This result indicated that this concentration of ouabain did not adversely affect the thalli, and further experiments could be carried out. The F_v_/F_m_ decreased significantly under hypersaline treatment (100‰, 15 min) and decreased even further after adding ouabain (**Figure 2A**).

### 3.3 ROS and MDA contents in *N. haitanensis* thalli under hypersaline conditions and in the presence of ouabain

The H_2_O_2_ and ${\text{O}}_{2}^{-}$ contents in the thalli did not change markedly under 100‰ hypersaline treatment (**Figure 2B, C**), but the ${\text{O}}_{2}^{-}$ content increased significantly after the addition of ouabain to inhibit NhNKA (**Figure 2C**). The MDA content significantly increased under hypersaline stress, and then further increased after the addition of ouabain (**Figure 2D**).

### 3.4 Ion fluxes in *N. haitanensis* under hypersaline stress and in the presence of inhibitors

**Figure 3:**
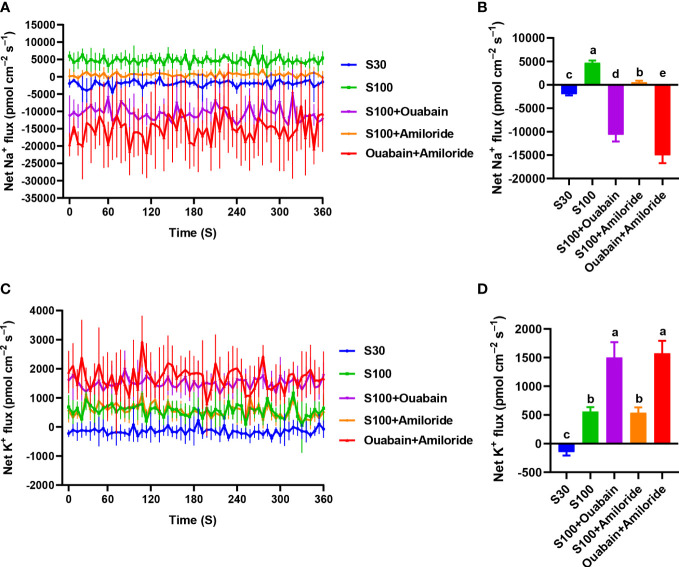
Effects of hypersaline (100‰, 15 min) and inhibitor treatments on net Na^+^ and K^+^ fluxes. **(A)** Na^+^ fluxes in the presence of ouabain and amiloride under hypersaline stress. **(B)** Mean rate of Na^+^ flux in thalli. **(C)** K^+^ fluxes in the presence of ouabain and amiloride under hypersaline stress. **(D)** Mean rate of K^+^ flux in thalli. S30: control salinity (30‰). S100: hypersaline stress (100‰). Each value is the mean of six individual thalli during 6 min measurements. Bars represent standard error of the mean (*n* = 6). Different letters indicate significant differences (*P<* 0.05) among treatments.

The Na^^+^^ flux had a mean rate of −1,984.67 pmol·cm^–2^·s^–1^ in the CK (**Figure 3B**). Hypersalinity (100‰, 15 min) resulted in significant efflux of Na^^+^^ (**Figure 3A**), with an average efflux rate of 4,725.58 pmol·cm^–2^·s^–1^ (**Figure 3B**). After the addition of ouabain (a Na^^+^^/K^^+^^-ATPase inhibitor) and amiloride (a SOS1 inhibitor), Na^^+^^ efflux was significantly restricted (**Figure 3A**), reaching −10,655.78 pmol·cm^–2^·s^–1^ and 548.83 pmol·cm^–2^·s^–1^, respectively (**Figure 3B**). When both inhibitors were added, Na^^+^^ efflux was markedly decreased (**Figure 3A**), with a mean efflux rate of −15,010.99 pmol·cm^–2^·s^–1^ (**Figure 3B**). In the CK, the average K^^+^^ influx rate was −146.60 pmol·cm^–2^·s^–1^ (**Figure 3D**). Hypersaline stress (100‰, 15 min) induced K^^+^^ efflux with a mean rate of 559.23 pmol·cm^–2^·s^–1^ (**Figure 3D**), while ouabain increased K^^+^^ efflux to a mean rate of 1,501.81 pmol·cm^–2^·s^–1^ (**Figure 3D**). Addition of amiloride, the K^^+^^ level (538.11 pmol·cm^–2^·s^–1^) had no significant difference compared to 100‰ hypersalinity (559.23 pmol·cm^–2^·s^–1^). When both inhibitors (amiloride and ouabain) were added, the K^^+^^ level (1,573.64 pmol·cm^–2^·s^–1^) had no significant difference compared to ouabain (1,501.81 pmol·cm^–2^·s^–1^) (**Figures 3C, D**).

### 3.5 Ion contents in *N. haitanensis* under hypersaline stress and in the presence of inhibitors

**Figure 4:**
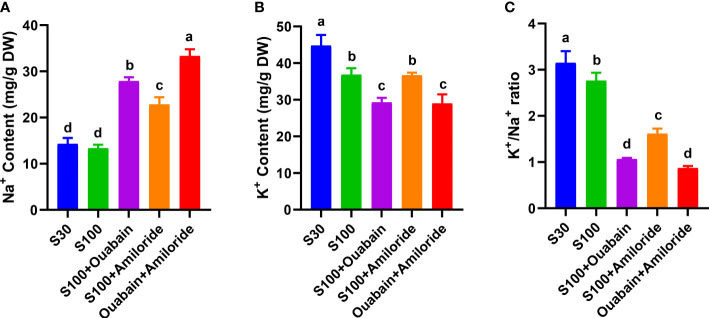
Effects of ouabain and amiloride on Na^+^ content **(A)**, K^+^ content **(B)**, and K^+^/Na^+^ ratio **(C)** in thalli under hypersaline stress (100‰, 15 min). DW: dry weight of thalli. S30: control salinity (30‰). S100: hypersaline stress (100‰). Values shown are average of three individual thalli. Bars represent the standard error of the mean (*n* = 3). Different letters indicate significant differences (*P<* 0.05) among treatments.

In normal seawater, the Na^^+^^ and K^^+^^ contents in *N. haitanensis* thalli were 14.28 mg/g dry weight (DW) and 44.78 mg/g DW, respectively (**Figures 4A, B**), and the K^^+^^/Na^^+^^ ratio was 3.15 (**Figure 4C**). Hypersalinity (100‰, 15 min) had no significant effect on Na^^+^^ content (**Figure 4A**), but significantly decreased the K^^+^^ content to 36.83 mg/g DW (**Figure 4B**), and significantly decreased the K^^+^^/Na^^+^^ ratio to 2.76 mg/g DW (**Figure 4C**). The addition of ouabain and amiloride under hypersaline conditions significantly increased the Na^^+^^ content (**Figure 4A**), and the Na^^+^^ level was significantly higher in the presence of ouabain (27.89 mg/g DW) than in the presence of amiloride (22.81 mg/g DW). The addition of both inhibitors under hypersaline conditions further increased the Na^^+^^ level in thalli to 33.33 mg/g DW (**Figure 4A**).

The addition of ouabain under hypersaline conditions significantly decreased the K^^+^^ content (29.26 mg/g DW), but the addition of amiloride had no significant effect (**Figure 4B**). Taking into account the Na^^+^^ content, the addition of ouabain and amiloride significantly decreased the K^^+^^/Na^^+^^ ratio to 1.06 and 1.61, respectively. The addition of both inhibitors together did not further decrease the K^^+^^/Na^^+^^ ratio compared with the addition of ouabain alone (**Figure 4C**).

## 4 Discussion

A high Na^^+^^ concentration induced by salt stress disrupts cellular K^^+^^/Na^^+^^ homeostasis, and adversely affects plant survival (Deinlein et al., 2014; Keisham et al., 2018; Yang and Guo, 2018). Therefore, timely and effective Na^^+^^ efflux is critical for the salt tolerance of plants. In this study, at 100‰ salinity treatment neither affected the Na^^+^^ content (**Figure 4A**) nor the H_2_O_2_ and ${\text{O}}_{2}^{-}$ contents in the thalli of *N. haitanensis*. This result suggests that the Na^^+^^ efflux system of *N. haitanensis* was able to operate effectively under these conditions to remove excess Na^^+^^ (**Figures 3A, B**). In addition to the SOS-like anti-transporter system, *Neoporphyra*/*Neopyropia* have Na^^+^^/K^^+^^-ATPases that are homologous to animal Na^^+^^/K^^+^^-ATPases (Kishimoto et al., 2013; Brawley et al., 2017). We found that the transcript level and enzyme activity of Na^^+^^/K^^+^^-ATPase were increased under hypersaline stress in the thalli of *N. haitanensis*. The addition of ouabain to inhibit Na^^+^^/K^^+^^-ATPase resulted in large-scale accumulation of Na^^+^^ in the cells (**Figure 4A**), leading to a significant decrease in the F_v_/F_m_ (**Figure 2A**) and increase in the ${\text{O}}_{2}^{-}$ content (**Figure 2C**) in thalli. Excessive ROS induced by Na^^+^^ can damage cellular components, leading to the accumulation of harmful substances such as lipid peroxidation products, including MDA (Wang et al., 2004). Therefore, these findings suggest that Na^^+^^/K^^+^^-ATPase is crucial for the response of *N. haitanensis* to salt stress.

Na^^+^^/K^^+^^-ATPase uses energy from ATP hydrolysis to expel three Na^^+^^ ions from the cell interior in exchange for two K^^+^^ ions entering the cell (Scheiner-Bobis, 2002). The plant membrane H^^+^^-ATPase hydrolyzes ATP to generate energy and pumps H^^+^^ out of the cytoplasm to generate an electrochemical gradient, which drives SOS1 to exclude Na^^+^^ (Yuan et al., 2016; Keisham et al., 2018). In the present study, the transcript level of SOS1 was also increased in the very early phase of hypersaline stress (**Figure 1D**), and the SOS1 inhibitor (amiloride) significantly blocked Na^^+^^ efflux (**Figures 3A, B**), resulting in an increase in Na^^+^^ content (**Figure 4A**) and a significant decrease in the K^^+^^/Na^^+^^ ratio in the thalli of *N. haitanensis* (**Figure 4C**). This finding suggests that under hypersaline stress, SOS1 can avoid Na^^+^^ toxicity to a certain extent by actively expelling Na^^+^^, as reported elsewhere (Chen et al., 2019). Although both amiloride and ouabain inhibited Na^^+^^ efflux under hypersaline stress, the inhibitory effect of ouabain was stronger than that of amiloride (**Figure 3B**), so the Na^^+^^ content was higher under ouabain treatment (**Figure 4A**). These results suggest that Na^^+^^/K^^+^^-ATPase has a larger Na^^+^^ excretion capacity, it may cooperate with SOS1 to regulate Na^^+^^ efflux in *N. haitanensis*.

Another negative effect of salt stress is the leakage of K^^+^^, because this ion plays an important role in enzyme activation, protein synthesis, stabilization of osmotic pressure, and maintenance of cytoplasmic pH homeostasis (Zhao et al., 2021). Our results and those of previous studies (Chen et al., 2019; Wang et al., 2020) show that the thalli of *N. haitanensis* have a strong capacity to maintain K^^+^^ levels under salt stress. The K^^+^^ content was nearly 37 mg/g DW in the thalli exposed to 100‰ salt stress (**Figure 4B**), only 18% lower than that in the CK. In the present study, ouabain treatment resulted in severe K^^+^^ leakage from the thalli (**Figures 3C, D**). In addition, the K^^+^^ content was significantly reduced from 37 mg/g DW under 100‰ salt stress to 29 mg/g DW after adding ouabain (**Figure 4B**). This suggested that Na^^+^^/K^^+^^-ATPase plays a significant role in the process of maintaining K^^+^^ in the thalli of *N. haitanensis* under hypersaline stress. Na^^+^^/K^^+^^-ATPase can combine with Na^^+^^ to promote ATP hydrolysis. The phosphorylation of an aspartic acid residue in Na^^+^^/K^^+^^-ATPase causes a conformational change that reduces its affinity for Na^^+^^, resulting in Na^^+^^ being pumped out of the cell. Meanwhile, extracellular K^^+^^ binds to Na^^+^^/K^^+^^-ATPase, and its subsequent dephosphorylation restores its conformation so that K^^+^^ is pumped back into the cell to complete the whole cycle (Kanai et al., 2021). In this way, the rapid uptake of K^^+^^ via Na^^+^^/K^^+^^-ATPase is the key pathway to preserve K^^+^^ levels under hypersaline stress, and is an important way in which Na^^+^^/K^^+^^-ATPase contributes to maintain K^^+^^/Na^^+^^ homeostasis of *N. haitanensis*.

## 5 Conclusion

This is the first study on the role of Na^^+^^/K^^+^^-ATPase in ionic homeostasis under salt stress in an intertidal macroalga. Hypersaline stress upregulated *NhNKA2* and increased Na^^+^^/K^^+^^-ATPase enzyme activity. This allowed the thalli to maintain K^^+^^/Na^^+^^ dynamic homeostasis under salt stress by expelling Na^^+^^ and maintaining K^^+^^ levels. When Na^^+^^/K^^+^^-ATPase activity was inhibited, the capacity to maintain higher K^^+^^/Na^^+^^ and scavenge ROS remarkably decreased. This suggests that Na^^+^^/K^^+^^-ATPase plays an indispensable role in the responses of macroalgae to salt stress.

## Data availability statement

The raw data supporting the conclusions of this article will be made available by the authors, without undue reservation.

## Author contributions

QC and WW performed the experiments and analyzed the data. WW and CX conceived and designed the experiments. YX, DJ, and CC analyzed the data and interpreted the results. QC and WW wrote the manuscript and prepared figures. WW and CX reviewed drafts of the manuscript. All authors contributed to the article and approved the submitted version.
